# Analytical and numerical solutions of the potential and electric field generated by different electrode arrays in a tumor tissue under electrotherapy

**DOI:** 10.1186/1475-925X-10-85

**Published:** 2011-09-24

**Authors:** Ana E Bergues Pupo, Juan Bory Reyes, Luis E Bergues Cabrales, Jesús M Bergues Cabrales

**Affiliations:** 1 Departamento de Física, Facultad de Ciencias Naturales, Universidad de Oriente, Patricio Lumumba s/n, Santiago de Cuba 90500, Cuba; 2 Departamento de Matemática, Facultad de Matemática y Computación, Universidad de Oriente, Patricio Lumumba s/n, Santiago de Cuba 90500, Cuba; 3 Departamento de Investigaciones, Centro Nacional de Electromagnetismo Aplicado, Universidad de Oriente, Ave. Las Américas s/n, Santiago de Cuba 90400, Cuba; 4 Computer Engineering Faculty, San Jorge University, Walqa Technological Park, Félix de Azara Building, 330a National Road, km 556, 22197. Cuarte, Spain; 5 Pharmacy Department, Faculty of Health Sciences, San Jorge University. Autov. A-23 Zaragoza-Huesca, km. 510, 50830 Villanueva de Gállego, Zaragoza, Spain

**Keywords:** Electrotherapy, Electric field, Tumor

## Abstract

**Background:**

Electrotherapy is a relatively well established and efficient method of tumor treatment. In this paper we focus on analytical and numerical calculations of the potential and electric field distributions inside a tumor tissue in a two-dimensional model (2D-model) generated by means of electrode arrays with shapes of different conic sections (ellipse, parabola and hyperbola).

**Methods:**

Analytical calculations of the potential and electric field distributions based on 2D-models for different electrode arrays are performed by solving the Laplace equation, meanwhile the numerical solution is solved by means of finite element method in two dimensions.

**Results:**

Both analytical and numerical solutions reveal significant differences between the electric field distributions generated by electrode arrays with shapes of circle and different conic sections (elliptic, parabolic and hyperbolic). Electrode arrays with circular, elliptical and hyperbolic shapes have the advantage of concentrating the electric field lines in the tumor.

**Conclusion:**

The mathematical approach presented in this study provides a useful tool for the design of electrode arrays with different shapes of conic sections by means of the use of the unifying principle. At the same time, we verify the good correspondence between the analytical and numerical solutions for the potential and electric field distributions generated by the electrode array with different conic sections.

## Background

Electrotherapy is the use of electrical energy as a medical treatment and it was introduced to destroy solid tumors at the end of nineteenth century. Many physicians have successfully used this therapy, also known as electrochemical tumor therapy, Galvanotherapy and electro-cancer treatment, as a standalone treatment in thousands of cases, with some truly spectacular results [1-4]. Electrotherapy of a low-level direct current is used to treat the cancer (target tissue) through two or more platinum (platinum-iridium 90/10, stainless steel) electrodes placed in or near the malignant tumor. In this therapy, two modes are used with similar results: voltage mode (voltage keeps constant and direct current intensity varies due to changes in the tumor resistance) and current mode (direct current intensity keeps constant for voltage variations because the tumor resistance is altered). In both modes, the tumor electrical resistance variations may be explained by different bioeffects induced in due to the application of this therapy.

The voltage mode produces less pain in the patient than the one induced for the current mode. The voltage range usually used is 6 to 12 V, the electric quantity often is 80 to 100 coulombs and the time needed to deliver this quantity is 20 to 120 minutes, in dependence of consistency, size and type of solid tumor. Permanent tissue damages are observed for voltage values equal and higher than 6 V and convenient distributions of electrodes in the tumor, as shown in our current clinical trial (results not shown) and [2-4]. As a result of these studies, 6 V may be considered as an irreversible threshold.

The clinical results carried out up to now reveal that, in both modes, electrotherapy is safe, effective, inexpensive, and induces minimal adverse effects in the organism. Also, it can be applied when the conventional methods (surgery, radiotherapy, chemotherapy and immunotherapy) fail. This anti-tumor therapy has not yet been universally accepted because two main reasons: 1) its antitumor mechanism is not fully understood and 2) it is not standardized [2-4]. The first reason is justified by the diversity of underlying antitumor mechanisms, such as: change of pH [5], immune system stimulation [2,4,6], lost of tissue water for electro-osmosis [7], the combined action of the toxic products from electrochemical reactions (fundamentally those in which reactive oxygen species are involved) and immune system stimulation [8], and the increase of the expression of dihydronicotinamide adenine dinucleotide phosphate dehydrogenase (NADPH) oxidase subunits-derived reactive oxygen species, which subsequently induces apoptosis of oral mucosa cancer cells [9], among others. In spite of this, the underlying mechanisms more widely accepted are the changes of pH and the toxic products from the electrochemical reactions. These changes are justified because the regions around the anode and cathode become highly acidic (pH ≤ 3) and highly basic (pH ≥ 10), respectively, when electrotherapy is applied to the tumor area [2-4]. Although Li et al. demonstrated that at the tumor center and areas far from the electrodes the pH is not modified and its value is similar to that measured in the unperturbed tumors (pH varies between 6 and 7) [10]. In a more recent work, Turjanski et al. demonstrated experimentally and theoretically that pH fronts spread in space and time. In particular, between electrodes, two pH fronts evolve expanding towards each other until collision [5]. The second reason is explained by the fact that the dosage guideline is arbitrary and dose-response relationships are not established. Also, different electrode placements are used however, optimal electrode distribution has not been determined. Electrotherapy standardization from the experimental point of view is complex, cumbersome, requires excessive handling of animals, and expensive resources and time. As a result, a natural and quick efficient way (few minutes) that may contribute to the standardization of this therapy is the mathematical modeling.

Electric field strength and its form of distribution, through electrodes play a decisive role in the electrotherapy effectiveness. The proposal for electrode arrays that efficiently distribute the electric field (electric current density) in a tumor and its surrounding healthy tissue is one of the most stimulating problems in the electrotherapy-cancer theme because the tumor may significantly be destroyed with the minimum damage in the organism. Different studies reveal that the electric field (electric current density) spatial distribution in tumor and its surrounding healthy tissue strongly depends on the tumor size, electrodes array parameters (applied voltage on the electrodes, number, positioning, size, shape, and polarity of them) and the electric field orientation [11-16]. Also, these distributions depend explicitly on the difference of conductivities of both tissues [13,15,16]. The influence of some of these parameters has experimentally been verified [3,4,6,17-19] and used to compute the power density distribution [20] and to increase the anti-tumor synergism of this therapy by means of the combination of this therapy with the intra-tumor injected saline solution, in agreement with previous results [3,4,21]. The good correspondence between the electric field spatial patterns obtained by experimental and theoretically ways has been demonstrated by Šersa et al. by means of the electric current density imaging technique [18]. Also, the influence of the ratio between direct current applied to the tumor and that distributed in it has been included in the Modified Gompertz equation [22].

In previous studies have been showed the two-dimensional (2D) analytical and numerical expressions for the potential and electric field generated by electrode arrays with circular [11,13] and elliptical [14,15] shapes. Jiménez et al. report three-dimensional (3D) analytical expressions to calculate the electric current densities in the tumor and its surrounding healthy tissue [16]. It has been reported that electric field (electric current density) inside the tumor increases with the increase of the tumor conductivity respect to that of its surrounding healthy tissue and when all electrodes are completely inserted in tumor [15,16]. These electric field (electric current density) spatial patterns and the conductivities in both tissues may be experimentally measured by means of different imaging techniques [18,23-30].

At present, several researchers have attempted to construct three-dimensional anatomical models for tissues by means of the finite-element method; however, an exact realistic tissue model is very difficult to establish from a computational point of view because it requires a precise knowledge of the electric and physiologic properties of both tissues. These electrical properties are the electrical conductivity, electrical permittivity, among others, whereas, the physiological properties are the type, heterogeneity, size, shape, composition, structure, consistency and water content of the tissue.

An aspect not widely discussed in the specialized literature is the knowledge of how the shape of electrode array affects the potential, electric field and electric current density distributions in order to improve the electrotherapy effectiveness. A significant effort is required to comprehend this problem because the exact shapes of different electrode arrays are usually not given, in spite of the existence of mathematical approaches [11-16] and imaging techniques [18,23-30]. Consequently, there exists a less exhaustive discussion of the comparison between these types of electrode arrays, in spite of the intent of some researchers of evaluating specific electrode configurations [1-4,6,17-19,31]. Precisely, the aim of this paper is to extend the results of Dev et al. [11], Čorović et al. [13] and Aguilera et al. [14,15] to electrode arrays with different shapes of conic sections (ellipse, parabola and hyperbola). For this purpose, we use the unifying principle for the conic sections and the analytical and numerical solutions. The potential and electric field distributions generated for each different conic section are compared.

## Methods

### Analytical calculations

Dev et al. [11], Čorović et al. [13] and Aguilera et al. [14,15] show, for the electrostatic problem, that the analytical solution in 2D for the potential and electric field around the needle electrodes can be obtained by solving Laplace equation, if the needle penetration depth is larger than the distance between the electrodes. It is worth noting that because any complex analytic function Φ(z), where z = x + iy, in a given region, is a solution of the Laplace equation

(1) $$\Delta \Phi \left(z\right)=0,$$

then its real part function, denoted by Re (Φ(z)), is also solution at the same region.

In Equation 1, Φ(z) is the potential that can be written as a sum of multi-poles of all electrodes [11]. The higher terms in multipole series are neglected with respect to the leading terms of all electrodes if the distance between electrodes is larger than the electrode radius. As a result, we can use the first term of this sum (lead order approximation, Φ^0^(z)) to calculate the electric field strength in lead order approximation, named E^0^(z) by means of ▽Φ^0^(z). The details for the calculations of Φ^0^(z) and E^0^(z) are reported in [11,13-15] and their analytical expressions are given by

(2) Φ0z= ∑n=1NCn lnaz-zn,

(3) $$E^{0}\left(z\right)=\overset{N}{\underset{n=1}{\sum }}C_{n}\left(\frac{a}{z-z_{n}}\right),$$

where N represents the total number of electrodes placed on the array and z is the position of the point where the calculations are made. a is the electrode radius and d the smallest distance between two consecutive electrodes with alternate polarities. $z_{n}=r_{n}e^{i\phi_{n}}$ is the position of the n-th electrode in the array. The coefficients C_n _in (3) are calculated from the boundary conditions of the electrodes and given in [11,13-15]. In Equation (2), a constant term is added if the number of electrodes is odd in order to satisfy conservation of the current, as shown in [13].

**Figure 1:**
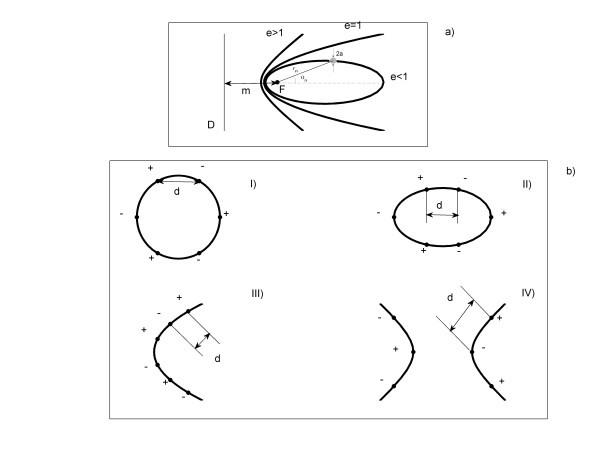
**Electrodes configurations**. (a) Conic sections: ellipse (e < 1), parabola (e = 1) and hyperbola (e > 1). (b) Electrodes array with shape of circle (I), ellipse (II), parabola (III) and hyperbola (IV). F, D, a, d, m, r_n _and θ_n _are defined in the text (see Method section).

The equations in polar coordinates for the conic sections (ellipse, parabola and hyperbola) may be obtained by using the unifying principle for the electrode position z_n_. In this way, r_n _can easily be shown to have a general expression in polar coordinates (common in form of the three curves) if the origin of coordinates is located in the conic focus, given by

(4) $$r_{n}=\frac{me}{1\pm e\cos\theta_{n}},$$

where m is the distance between the focus (F) and the directrix line (D), as shown in Figure 1a. The straight line passing through F and perpendicular to D is assumed to be the prime direction, from which the angles are measured. r_n _y θ_n _are the polar coordinates of the n-th electrode (with the origin on the point F). The parameter e is the conic eccentricity that distinguishes the type of conic section: e < 1 (the locus is an ellipse); e = 1 (the locus is a parabola) and e > 1 (the locus is a hyperbola). Although the unifying principle for the conic sections allows the possibility to obtain the polar equations of all three curves (4), it should be remarked that in the case of hyperbola this equation represents only one of its branches (that whose focus is at the origin), rather than the entire curve. The plus sign corresponds to the left branch of the hyperbola.

Figure 1b shows electrode arrays with different shapes: circle (Configuration I, e = 0), ellipse (Configuration II, e = 0.6), parabola (Configuration III, e = 1) or hyperbola (Configuration IV, e = 2). The expressions for the potential and electric field intensity obtained for Configuration 1 are explicitly given in [11,13] (for r_n _= b: b is the circle radius) and [14,15] (for r_n _= b_1 _= b_2 _= b: b_1 _and b_2 _are the major and minor radius of the ellipse, respectively). It is important to point out that the expressions reported in [11,13-15] are referred to the origin of coordinates in the conic center. Two additional electrode arrays are used: one elliptical with e = 0.45 (Configuration II-1) and another hyperbolic with e = 3 (Configuration IV-1), keeping constant parameter m in order to evaluate the influence of parameter e. Table 1 shows the parameters e and m of each one of these configurations.

**Table 1:** Values of eccentricity (e) and distance between the focus and the directrix (m)

Types of electrode configurations	Parameters of the electrode array	
	
	e	m (mm)
Configuration I (circle)	0	-
Configuration II (ellipse)	0.6	7
Configuration II-1 (ellipse)	0.45	7
Configuration III (parabola)	1	7
Configuration IV (hyperbola)	2	7
Configuration IV-1 (hyperbola)	3	7

Following the ideas of Čorović et al. [13], we assume that the ratio U/d = 0.115 V/mm is a constant, where U is the potential difference between two nearest electrodes. As a result, the potential in each electrode (V_0_) is ± V_0 _= U/2. Table 2 shows the values of d, U and V_0 _for each one of these configurations. We fix the electrode radius (a = 0.215 mm) and the number of the electrodes (six electrodes with alternate polarities). The angular positions of these six electrodes (θ), with respect to the center of the conic, are fixed in θ = 0, 60, 120, 180, 240 and 300° (for the circle and the ellipse), and θ = 0, 45, 135, 180, 225 and 315° (for the hyperbola). In the case of the parabola, the angular positions are referred to vertex in θ = 60, 65, 75, 285, 295 and 300°. In order to calculate the equation (4), these positions are transformed to those with respect to the focus F.

**Table 2:** Values of the potential (U) and distance between two closer electrodes (d) for each electrode configuration

Types of electrode configurations	d (mm)	U (V)	± V_0 _(V)
Configuration I (circle)	5.00	0.575	0.288
Configuration II (ellipse)	5.50	0.633	0.316
Configuration II-1 (ellipse)	3.61	0.415	0.208
Configuration III (parabola)	2.25	0.259	0.129
Configuration IV (hyperbola)	5.81	0.668	0.334
Configuration IV-1 (hyperbola)	2.81	0.323	0.161

U/d is a ratio constant (0.115 V/mm).

### Numerical Calculations

Numerical calculations are performed by using a finite element method for each electrode array in 2D. The electrodes are placed inside a rectangle representing a homogeneous tissue having a constant conductivity. For the analytical and numerical calculations, the electrodes are completely inserted in the tumor because the higher electric field strength (electric current density) is induced in it with the minimum damage in the surrounding healthy tissue [15,16].

A constant voltage is assigned to the boundary representing the electrodes surface. Isolating boundary conditions are assigned to the outer boundaries of the rectangle. The dimension of the outer square is 20 mm > 2d in all models, since 2d is the error due to the finite size of the model is negligible. The values of the parameters e, m, a, electrode potential and angular position of each electrode are the same as those used for the analytical calculations. Model geometries are meshed by triangular finite elements. The final mesh is obtained by an adaptive method using a relative tolerance criterion of 0.001.

The maximum (E_max_, in V/mm), minimum (E_min_, in V/mm) and norm (EE, in V/mm) values are used to quantify the differences between the electric field distributions generated for each electrodes array. E_max _and E_min _represent the local characterization of the electric field and EE is the global characterization of it. Indeed, EE is the sum of the local electric field intensity over all points in the target tissue, given by

(5) $$EE=\sqrt{\sum_{k=1}^{p}{\left|E_{k}\right|}^{2}}.$$

where E_k _is the local electric field intensity in each point k (k = 1, ..., p) and p is the total number of points in the target tissue (p = 32 248).

Finite element method and the expressions (2-5) are implemented in the MATLAB software, version R2011a (License number: 625596. San Jorge University, Spain). The analytical and numerical calculations are performed on a personal computer Intel Pentium 4, dual-core processor 2.16 GHz CPU and 4 GB RAM. Each calculation takes approximately one minute.

## Results

**Figure 2:**
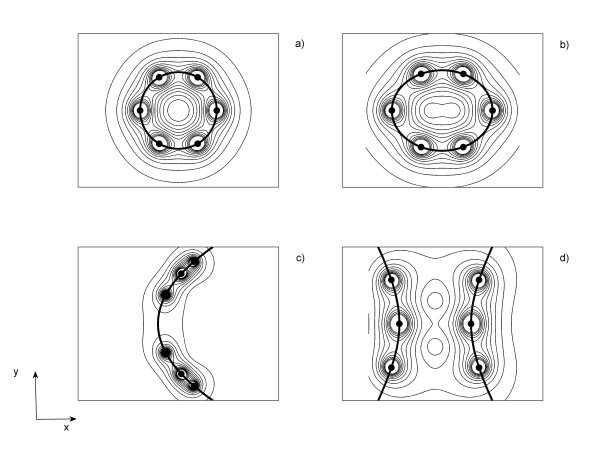
**Electric field spatial patterns**. Analytical results of the electric field distributions for the electrodes configurations with shapes of a) circle, b) ellipse, c) parabola and d) hyperbola defined in Fig. 1b.

Figure 2 shows the electric field distributions for the electrode array with different shapes: circle (Figure 2a), ellipse (Figure 2b), parabola (Figure 2c) and hyperbola (Figure 2d). The isolines are drawn for the electric field values from 0 to 0.115 V/mm with a constant step of 0.01 V/mm. It illustrates how the electric field distributions in the tissue depend on the shape of the electrodes array and that the highest electric field strengths are obtained in the neighborhood of the electrodes. The electric field strength falls even more rapidly towards the tumor edges in the perpendicular direction to the plane in which the electrodes are. Also, these figures reveal that the electric field between the electrodes is non-uniform whereas in the central region is uniform.

Table 3 shows E_max_, E_min _and EE values for each one of the configurations above mentioned. These three quantities are calculated over all nodes within the work region. This table and Figure 2 evidence that Configurations I, II and IV concentrate more the electric field lines in the target tissue and show the higher values of these quantities. In contrast, Configuration III concentrates less these lines in it and shows the smallest values of EE and E_max_. This configuration concentrates the electric field lines mainly around the electrodes.

**Table 3:** Values of the maximum electric field strength (E_max_), minimum electric field strength (E_min_) and electric field norm (EE) for each electrode configuration

Types of electrode configurations	E_max_ (V/mm)	E_min_ (V/mm)	EE (V/mm)
Configuration I (circle)	37.5855	0.0000	44.9545
Configuration II (ellipse)	37.8268	0.0002	39.7075
Configuration II-1 (ellipse)	8.3062	0.0001	17.6613
Configuration III (parabola)	3.2166	0.0000	10.0745
Configuration IV (hyperbola)	30.1479	0.0002	32.7382
Configuration IV-1 (hyperbola)	6.9787	0.0000	16.1356

The comparison between the electrode elliptical arrays (Configurations II and II-1) and electrode hyperbolic arrays (Configurations IV and IV-1) evidences that there exist differences in the electric field distributions when parameter e varies, keeping constant parameter m, the type of electrode configuration, the angular position and the polarity of the electrodes. It is easy to check that electric field distribution generated for each conic section changes when the electrode polarity and values of the parameter m are varied (results not shown).

**Figure 3:**
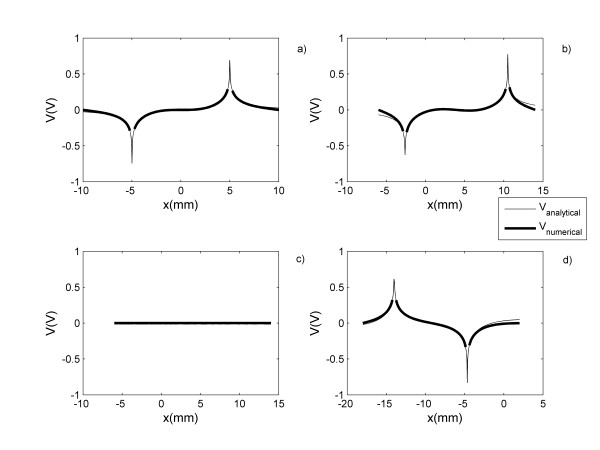
**Comparison of the analytical and the numerical solutions**. The analytical and the numerical solutions of the electric potential distribution along y = 0 direction generated by electrode arrays with shapes of a) circle, b) ellipse, c) parabola and d) hyperbola defined in Fig. 1b.

**Figure 4:**
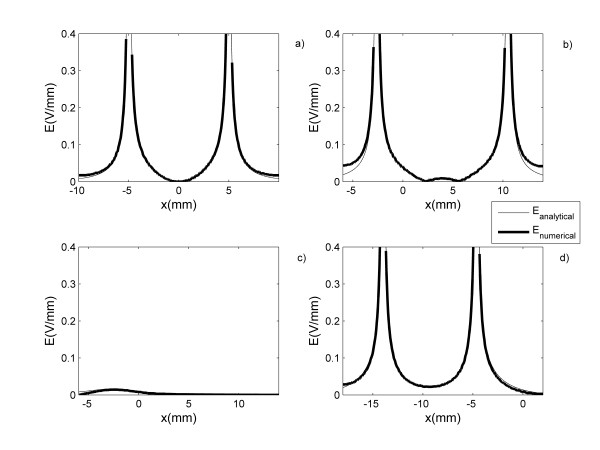
**Comparison of the analytical and the numerical solutions**. The analytical and the numerical solutions of the electric field distribution along y = 0 direction generated by electrode arrays with shapes of a) circle, b) ellipse, c) parabola and d) hyperbola defined in Fig. 1b.

The analytical results are validated by the numerical calculations for each electrodes configuration. Comparison of the numerical and analytical results are carried out by plotting the potential (Figure 3) and electric field (Figure 4) along the y = 0 direction. These figures show the behavior of these two physical quantities for the electrode arrays with circular (Figures 3,4a), elliptical (Figures 3,4b), parabolic (Figures 3,4c) and hyperbolic (Figures 3,4d) shapes. Figures 3 and 4 reveal a good agreement between the numerical and analytical results inside each electrode array. Also, the numerical calculations reveal similar electric field distributions for each conic section than those shown with the analytical calculations. However, in the outer region, the discrepancy between both solutions increases with the increase |x|. Similar results are observed in any directions.

## Discussion

In this paper we do not pretend to discuss whether the analytical solution is better than numerical one or vice versa. The results demonstrate that the analytical calculations shown in [11,13-15] can be extended also to the electrode configurations used in this paper. This mathematical approach is simple and constitutes a rapid and simple method for visualizing both potential and electric field distributions inside the target tissue without using special software for numerical modeling. That is why, we use the analytical method to know the exact dependence of the potential and electric field distributions in function of the electrodes array parameters. The validity of this method from the mathematical point of view is verified by the good agreement between analytical and numerical solutions for each electrodes configuration in the area between the electrodes. From the biological point of view, this validity may be reinforced by means of an *in vivo *(*ex vivo*) tissue model.

We use 2D numerical and analytical models in order to compare the potential and electric field strength, for different electrode configurations, in the central plane of a more general 3D model. The 2D results are a good approximation of local electric field distribution in 3D models for needle electrodes since these are usually long and deeply inserted in tissue, as is reported in [13]. Also, these results evidence that the electric field distributions depend markedly on the shape of electrodes array with respect to target tissue. This is possible by means of the use of the unifying principle for the conic sections that allows the knowledge of the exact geometry of the electrode array in a very clever way and therefore U/d ratio facilities the comparison between the different studies reported. This ratio is an approximation widely used to estimate the electric field intensity inside the tumor.

E_max_, E_min _and EE values may be useful to propose electrode configurations more feasible for tumor treatment. Configurations I, II and IV concentrate more the electric field lines in the target tissue between the electrodes. As a consequence, these may be suggested for the solid tumors treatment with electrotherapy and other electric field based therapies, as electrochemotherapy and irreversible tissue ablation. For this, we should keep in mind that electric field strength should be above a certain irreversible threshold value of the electric field in order to cause permanent damages on the target tissue leading to its partial or complete destruction. However, it should not be exposed to excessively high electric field to avoid damages to the surrounding healthy tissue.

At first sight, Configuration III is un-useful for the solid tumors treatment if we keep in mind that it concentrates less the electric field lines in the tumor and shows low values of EE and E_max _(10 times lower than that obtained by Configurations II and IV). We have observed that the tumor complete remission and the conversion of an inoperable tumor in operable (patients with breast cancer) are reached, independently of the tumor histological variety for voltage strengths below 6 V. In this case, we make a convenient distribution of the electrodes in the tumor combined with the intra-tumor injected saline solution.

A potential clinical application of Configuration III may be in the selective treatment of the tumor-healthy tissue interface (or tumor border), which is a complex region due to the simultaneous presence of both cancerous and healthy cells and other cellular components.

This interface is rich in blood and lymphatic vessels, in dependence of the tumor type, in addition to the existence of high sialic and lactic acid concentrations, fact that may indicate that this tumor region has high conductivity. In this case, it is not required high electric field strength. The knowledge of this interface may be interest for the therapist and an indicator of the difference between the tumor and its surrounding healthy tissue, aspects which should be considered in the therapeutic planning before treatment. This allows an adequate insertion and distribution of the electrodes inside and/or at the tumor border, in dependence of the electrodes of the electrodes configuration type in agreement with other studies [11,13-16]. Hence, we should keep in mind that the surrounding healthy tissue is affected by the electric field (electric current density) when the electrodes are inserted outside and/or at border of the tumor, being more marked when the tumor differentiates more than its surrounding healthy tissue, as previously reported by other authors [13,15].

Also, Configuration III may be used for cancer treatment if we use symmetric parabolic configurations (similarly as for Configuration IV) and/or combining it with other pieces of different conic sections and the electrode arrays actually used. From the electrode configurations above mentioned, it is possible to propose other more complex electrode arrays: i) two elliptical pieces with different eccentricities; ii) one elliptical piecewise of eccentricity e with the parabola; iii) one elliptical piecewise of eccentricity given with one branch of the hyperbola; iv) the parabola with one branch of the hyperbola of eccentricity e; and vi) two branches of hyperbola with different eccentricities). For this, we fix the origin (vertex) in the focus of one piecewise the ellipse and hyperbola (parabola) and thus express the equation of the other piecewise of another conic section with respect to this frame of reference (origin) by means of a translation to the focus of the first conic. This allows the use of Configurations I, II, III and IV, though these have not been used in the preclinical and clinical studies.

The above mentioned is important in the therapeutic planning previous to the electrotherapy application because we may choose the polarity and positioning of the electrodes, as well as the shape of the electrodes array, which have a marked influence in the potential and electric field distributions. These electric field distributions generated for these electrode arrays may be experimentally verified by means of diverse imaging techniques as the Electric Current Density Imaging [18,27], Electrical Impedance Tomography [28], Magnetic Resonance Electrical Impedance Tomography [29], Magnetic Induction Tomography, Magnetoacoustic Tomography and Magnetoacoustic Tomography with Magnetic induction [30]. Also, for showing the plausibility of this mathematical approach, an *in vivo *model may be implemented in order to evaluate the influence of the parameters of these electrode arrays in the tumor growth kinetic, aspect that may be theoretically corroborated, as previously reported by Cabrales et al. [22].

**Figure 5:**
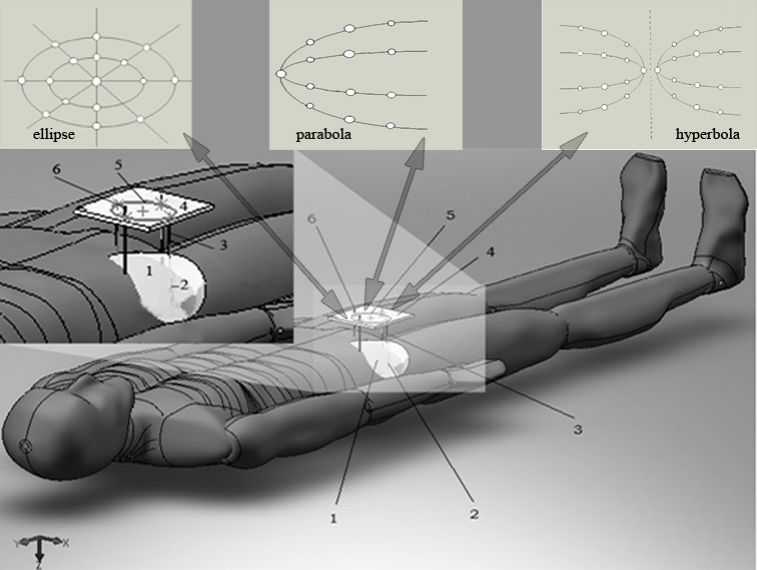
**Schematic representation of the electrodes insertion in clinical studies**. Electrodes inserted along of the deep tumor and distributed according to the conical section type (ellipse, parabola and hyperbola). Isometric projection of a solid tumor in the human body: (1) solid tumor, (2) electrode inside the tumor, (3) electrode inside the plastic cannulae, (4) board plastic with its printed conical section (5), and (6) the electrode edge that is connected to the direct current device.

We are not aware of the use of the conic sections in electrotherapy (electrochemotherapy and ablation therapy) for the cancer, but the use of these is feasible in the preclinical and clinical studies. In patients with cancer, these electrode configurations should be used in order to evaluate the safety (phase I of a clinical trial), adverse effects and toxicity (phase II of a clinical trial), and effectiveness (phase III of a clinical trial). In clinical studies, the electrodes insertion methodology for Configurations I, II, III and IV is similar to that used at present (electrodes inserted into the base perpendicular to the tumor long axis) [1-6,17,19]. The essential steps of this methodology are:

1. The tumor size is determined by clinic and/or any imaging techniques (ultrasound, Computer Tomography or Imaging Nuclear Magnetic Resonance). Plastic cannulae with style are inserted, through holes (printed in a plastic board and distributed in a family of conic sections that completely cover the tumor size), as shown in Figure 5 for an electrode elliptical array (isometric projection). This is also valid for electrode arrays with other shapes (circle, parabola and hyperbola).

2. The styles are withdrawn and the electrodes are inserted in the tumor mass through the cannulae to ensure that the electric field will cover all the tumor mass when the voltage is applied to the electrodes (Figure 5). After insertion of the electrodes, the cannulae are withdrawn to the edge of normal tissue. This procedure guarantees that the electrodes are completely inserted into the solid tumor to maximize tumor destruction with the minimum damage in the organism. Finally, the electrodes are connected to the negative poles (the cathodes) of a custom built constant voltage (current) generator, and the other needles are connected to the positive poles (the anodes).

The results of this study suggest that different physical and chemical quantities, such as heat, temperature, pH fronts and electrochemical reactions around electrodes may be calculated from the electric field generated by electrode arrays with shapes of conical sections, which may contribute to the understanding of the electrotherapy antitumor mechanisms, as previously report other authors [5,10,18,20,32].

## Conclusion

In conclusion, the mathematical approach presented in this study is an extension of the works of Dev et al. [11], Čorović et al. [13] and Aguilera et al. [14,15] and constitutes a useful tool for the design of electrode arrays with different shapes of conic sections by means of the use the unifying principle. Also, there is a good correspondence between the analytical and numerical solutions for the potential and electric field distributions generated by the electrode array with different conic sections.

## Competing interests

The authors declare that they have no competing interests.

## Authors' contributions

JBR developed the mathematical idea on which this manuscript is based. All the computer simulations and results analysis were making by AEBP. LEBC and JMBC supervised this research and helped in the results analysis. Also, all authors read and approved the final version of the manuscript.
